# In Situ Measurement of Airborne Particle Concentration in a Real Dental Office: Implications for Disease Transmission

**DOI:** 10.3390/ijerph18178955

**Published:** 2021-08-25

**Authors:** Maryam Razavi, Zahid Ahmad Butt, Helen Chen, Zhongchao Tan

**Affiliations:** 1 Department of Mechanical and Mechatronics Engineering, University of Waterloo, Waterloo, ON N2L 3G1, Canada; m5razavi@uwaterloo.ca; 2 School of Public Health Sciences, University of Waterloo, Waterloo, ON N2L 3G1, Canada; zahid.butt@uwaterloo.ca (Z.A.B.); helen.chen@uwaterloo.ca (H.C.)

**Keywords:** dental aerosol, particle concentration, infection transmission, particle removal, and indoor air quality

## Abstract

Aerosols generated during dental procedures are one of the most significant routes for infection transmission and are particularly relevant now in the context of COVID-19 pandemic. This study aimed to assess the effectiveness of an indoor air purifier on dental aerosol dispersion in dental offices. The spread and removal of aerosol particles generated from a specific dental operation in a dental office are quantified for a single dental activity in the area near the generation and corner of the office. The effects of the air purifier, door condition, and particle sizes on the spread and removal of particles were investigated. The results show that, in the worst-case scenario, it takes 95 min for 0.5-μm particles to settle and that it takes a shorter time for the larger particles. The air purifier expedited the removal time at least 6.3 times faster than the case with no air purifier in the generation zone. Our results also indicate that particles may be transported from the source to the rest of the room even when the particle concentrations in the generation zone dropped back to the background. Therefore, it is inaccurate to conclude that indoor purifiers help reduce the transmission of COVID-19. Dental offices still need other methods to reduce the transmission of viruses.

## 1. Introduction

Dental professionals, staff, and patients in dental offices are exposed to aerosol droplets, particles, and pathogenic microorganisms in the saliva and blood of the infected patients. The infectious microorganisms transmitted from blood include HIV and hepatitis viruses and from saliva and nasopharyngeal secretions include pneumonic plague, Legionella pneumophila, tuberculosis, influenza viruses, herpes viruses, SARS virus (a form of coronavirus), pathogenic streptococci, and staphylococci [1,2]. Recently, the SARS-CoV-2 virus, which caused COVID-19, joined this list because studies show that dentists are at high risk of exposure to this virus, even more than nurses [3]. These infectious diseases, particularly COVID-19, could be transmitted from pre-symptomatic or asymptomatic patients to others even in the recovery phase [4]. Infectious microorganisms spread in dental offices via various routes [5]. These routes include direct contact with the body fluid or surfaces and instruments touched by an infected person, contact with the exhaled air from the infected person, and infection transmission through aerosols generated during the dental procedures [1,5,6,7].

Airborne transmission is the considerable route of disease transmission mostly associated with aerosol smaller than 5 μm in diameter, recognized by the World Health Organization (WHO) in healthcare settings [8]. Splatters are another potential source of infection. Splatters are a mixture of air, water, and solid substances [2]. As the water evaporates, the smaller splatters linger longer in the air. Exposure to high-concentration aerosols and splatters in a dental office increases the probability of infection transmission from person to person [1].

In addition to the disease transmission through aerosol, exposure to non-biological aerosol particles in the dental offices and laboratories adversely affects human health [2]. Particles generated during abrasive dental procedures and material treatment from various substances cause health issues owing to their fine size [9]. Hence, dental aerosols may count as contamination in indoor working environments [10]. Size and mobility are key factors for their penetration into the respiratory system and potential health risks [11]. There is a direct correlation between the respiratory system infections of dental personnel and the concentration of generated aerosols due to the dental procedures. Particles smaller than 2.5 micrometers (PM2.5) may deposit in the alveolar region of the respiratory system [12]. They can further enter the bloodstream, causing lung cancer, pulmonary and cardiovascular diseases, heart diseases, asthma, increased mortality, and so on [13,14].

According to the American Dental Association (ADA), the Centers for Disease Control and Prevention (CDC), and the Occupational Safety and Health Administration (OSHA), all the contaminated aerosol and splatters should be eliminated as much as possible from the air in the dental offices and related laboratories [1,15,16]. It is necessary not only for the protection of people in the dental offices but also for the control of disease outbreaks.

Strict and effective infection control protocols are highly required to fight COVID-19 in dentaries [5] as well as other indoor spaces because of the potential for airborne transmission of SARS-CoV-2 (the coronavirus that causes COVID-19) through aerosols [17]. General preventive measures and dental practice recommendations have been developed during the COVID-19 pandemic [18,19]. Recent guidelines by WHO recommend delaying routine, non-essential oral health care [20]. Moreover, the Ontario Dental Association guidelines require three hours between two patients during the first wave of COVID-19 pandemic [21]. These guidelines pose a significant challenge to the dental business due to reduced or no patient visits. Therefore, systematic research is needed for the decision makers to develop alternative preventive measures and recommendations.

Protection methods are constantly emphasized in the guidelines. Multiple approaches may help reduce the transmission of infectious diseases. The use of personal protections, such as facemasks, gloves, and goggles, are recommended to reduce the exposure of dental staff to aerosol; however, facemasks are not 100% effective [9,22]. Measurements show a very high concentration of particles $9.7\times 10^{5}/{\mathrm{cm}}^{3}$ even behind surgical masks [9]. Rubber dams and conventional dental suctions (CDS) can protect patients [1,9,23]; however, their uses are limited to certain dental operations [1,9]. Moreover, conventional dental suctions are not very effective in eliminating sub-micron particles [9]. A high-efficiency particulate air (HEPA) filter and the use of ultraviolet (UV) chambers in the ventilation system are other protective methods that are effective after threats have become airborne and spread to the room [1]. A high-volume evacuator (HVE) is a suction device that can reduce the aerosol by more than 90% [1]. It removes a large volume of air within a short duration, and the evacuation system can remove a volume of air up to 100 cubic feet per minute [24]. Although the HVEs are recommended to reduce airborne contamination exposure, they have some limitations. Clinicians may experience the difficulty of handling the high vacuum pressure and blocking the view of the operator. Using HVEs might not be applicable for the operator without an assistant [1]. Furthermore, its performance depends on the volumetric rate of evacuation and particle-generation rate [1]. Recent COVID-19 outbreak has resulted in increased use of portable air purifiers in dental offices despite the scarcity of published research on their performances in dental offices [25,26]. Further research on the protective effectiveness of air purifiers in dental clinics was recommended [27]. The portable air purifiers can be located at the corners of the dental offices, and they cause much less inconvenience during dental operations than extra-oral high evacuators do. In addition, these portable air purifiers do not require modification to existing ventilation systems.

Despite the research on the measurement of number concentrations for micron [28,29] and nano-size particles [30,31,32] generated from dental procedures, to the best of our knowledge, no experimental research has been done on the spatial distribution and transport of airborne particles lingering in different parts of the office. The spatial distribution of them is one of the key considerations on health issues. The size of aerosol might be one of the most determinative parameters in their spatial distribution. Aerosol particles with a diameter of 50 μm remain suspended in the air for up to 30 min after their formation [33]; however, this time is obtained based on the calculations. Larger aerosol and splatters, which are heavier, will fall rapidly to the floor [2], while smaller particles may remain airborne much longer. The nature of the extensive surface area in dental offices may enhance the losses of particles onto various surfaces. Furthermore, research on the effects of air purifiers on the removal of aerosols is needed to develop guidelines and protocols to reduce waiting time between patients and ensure the safe operation of dental offices.

The objective of this study is to understand the temporal concentration distribution and removal of airborne particles (<0.5 to 20 µm) generated during a single dental activity of drilling to minimize the health issues associated with these particles. The remainder of this paper is presented as follows. Section 2 presents the material and study design of concentration measurements in the dental office. In Section 3.1, the number concentration distribution of particles under the effects of operating conditions during the generation is investigated. After the generation, the spatial and temporal change of particle concentrations distribution under the effects of operating conditions are investigated in Section 3.2 at the generation zone and Section 3.3 at the corner of the office. We determined the effectiveness of air purification as well as other effective mechanisms in the removal of the particles of various sizes. Finally, the study is summarized in Section 4. Results in this paper are deemed useful to the best practices for particle removal from dental offices.

## 2. Materials and Methods

### 2.1. Measurement Site and Instruments

**Figure 1:**
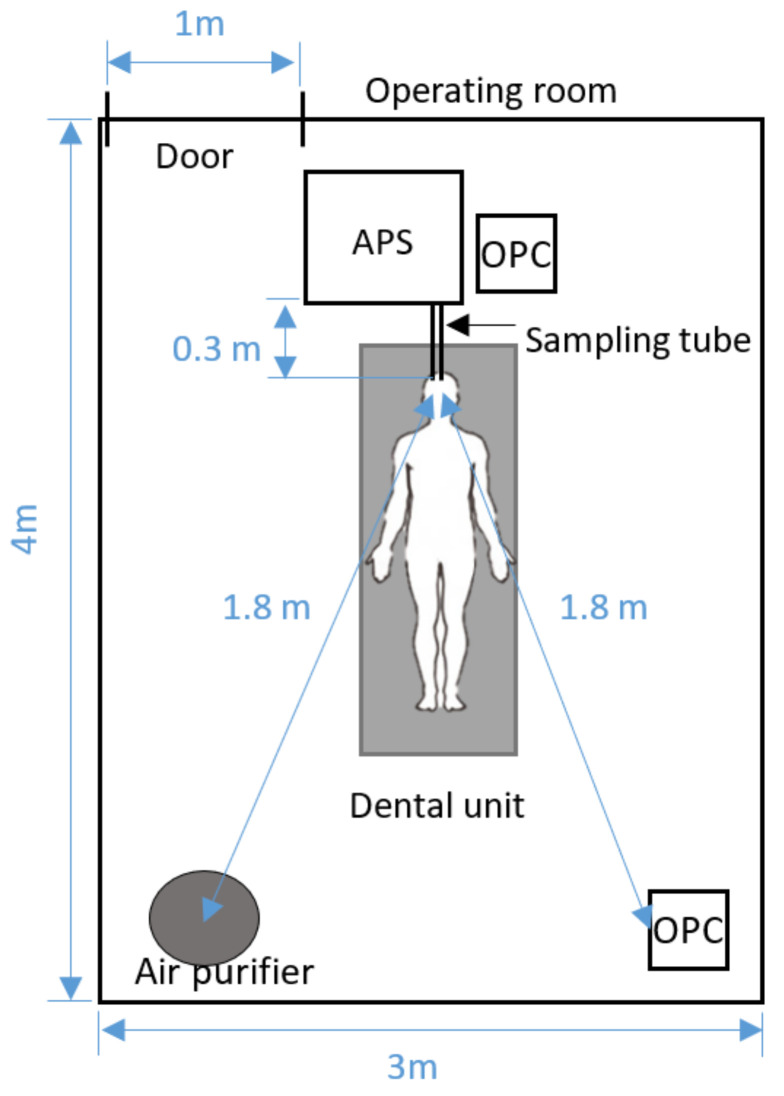
Schematic of the experimental setup.

The concentrations of micron and submicron particles were measured on 15 May 2020 in a dental operation room on the second floor of the dental clinic in Toronto, ON, Canada. Figure 1 shows the schematic of the operatory and layout of the instruments. This typical dental operatory room is 3 m wide, 3 m long, and 4 m high; it has one dental unit. These measurements are conducted in a typical dental operatory room. Further studies in different room layouts may provide more generalized conclusions. In this research, the ventilation system in the measured dental office was sensitive to the temperature and could not be controlled. Therefore, the mechanical ventilation system was off, and the window was closed throughout the test to create a relatively controlled environment for testing and focus on the worst-case scenarios. Although air circulation caused by ventilation decreases the removal rate of particles, it might facilitate the distribution of contaminated air throughout spaces [34]; therefore, it is not recommended. One of the focuses of this research is indoor air purification, in which the air purifier was running continuously in the room.

The number concentrations of particles were measured using an aerodynamic particle sizer spectrometer (APS, TSI 3321) and two optical particle counters (OPC, Handheld 3016, Lighthouse Worldwide Solutions Inc., USA). The APS took data every 5 min with 5 scans; each scan lasted 20 s; it can detect the particles in the range of 0.5–20 µm in diameter and those smaller than 0.5 µm. The APS was located on the left-hand side of the doctor to prevent any inconvenience for the doctor during dental operations. A stainless-steel sampling tube, which has a 0.635-cm inner diameter and is 0.3 m long, was connected to the inlet of the APS for sampling air 10 cm away from the operation area (i.e., the patient’s mouth). Both OPCs were running continuously. One OPC was located beside the APS, and another OPC was 1.8 m away from the source. Both OPCs report particles with diameters of 0.3, 0.5, 1, 2, 5, and 10 µm. The first OPC is calibrated against the APS.

### 2.2. Study Design of Dental Operation on Pig Jaw

**Figure 2:**
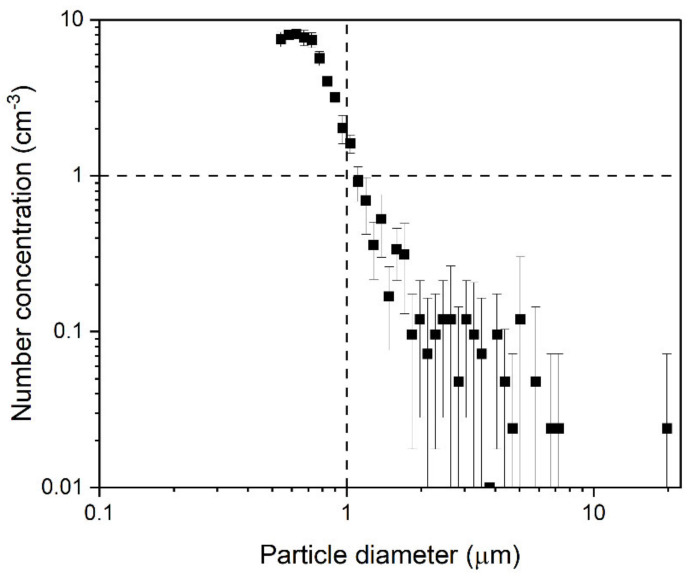
Background number concentration for 0.5–20 µm aerosols.

Before the operation, the room was unoccupied for 15 h before the background concentrations were measured at the generation zone without air purification. The measurement was conducted using APS for 30 min. The APS took data every 5 min with 5 scans; each scan lasted 20 s. The average value of all scans was reported with the standard deviation. Three people were present in the room during the measurement. Background concentration was also measured before each scenario, which was similar to background concentration measured at the beginning of the day. Figure 2, shows that all particles in the background air were less than 10/cm^3^, and those larger than 1 µm in diameter were less than 1/cm^3^.

At the beginning, particles were generated over 5 min of continuous drilling operation on a pig jaw using high-speed handpiece. The high-speed handpiece is operated with a range of 250,000–400,000 rpm by using compressed air [35].

Pig teeth are commonly used for dental studies because of similarities between the structure of human and pig enamel and dentin [36,37]. The particle number concentrations were measured during 5 min of continuous dental operation and afterward until the number concentrations reached the background. Then the airborne particle concentrations under six cases were measured. Table 1 shows the conditions of these cases. All particles were generated by drilling the pig jaw with a high-speed handpiece. Other factors considered include the door condition (open and close) and air purifier (on and off, airflow rate, starting time). The air purifier (surgically clean air, model: JADE, SCA5000C) was 1.8 m away from the generation zone. The air purifier unit has a HEPA Filter (remove particles), Activated Carbon Filter (absorbs odors and gases), Germicidal UV-C+, and Super Oxidizing Photocatalytic Nano-TiO2 system (killing airborne pathogen) and two Revitalizing Negative Ion generators (revitalize and refresh the air). The air purifier has a cylindrical shape, with a diameter of 30.5 cm and a height of 85 cm. The contaminated air enters the unit from the lateral surface and clean air exits from the ring shape top of the cylinder. The performance of the air purifier is tested with two fan speeds of 153 CFM and 312 CFM corresponding to air changes per hour (ACPH) of 7.23 and 14.73 in the tested room, respectively. The reported filtration efficiency of the air purifier filter by the supplier is >99.99% for 0.1–5-µm particles.

The particle concentrations were measured at two locations in the room: one is at the source, and another is at the corner of the room. These data allow us to analyze the spatial and temporal change of particle concentration distributions. The concentrations at the corner help determine the probable accumulation of particles where there is the least air circulation.

### 2.3. Study Design of Real Operation with the Patient

In the second part of this study, the number concentrations were measured by APS for three dental operations with real patients. The air ventilation system was blocked, and the door was closed; however, it was opened several times during the operations.

Table 2 summarizes the conditions for these three operations. The first and second operations were done in the large room 4×5×3 m (W×L×H). The sampling tube was 30 cm away from the patient’s head on the left-hand side of the doctor’s chair. The sampling tube was blocked several times by the doctor’s arm during the operation. In the first operation, the air purifier was running in periodic mode between low speed (153 CFM) and turbo speed (406 CFM), while it was off for the second and third operations. The third operation was conducted in a small room. The sampling tube was 30 cm away from the patient’s head. The sampling tube was located on the opposite side of the doctor to avoid blockage.

## 3. Results and Discussion

### 3.1. Particle Generation during Operation for Five Minutes

**Figure 3:**
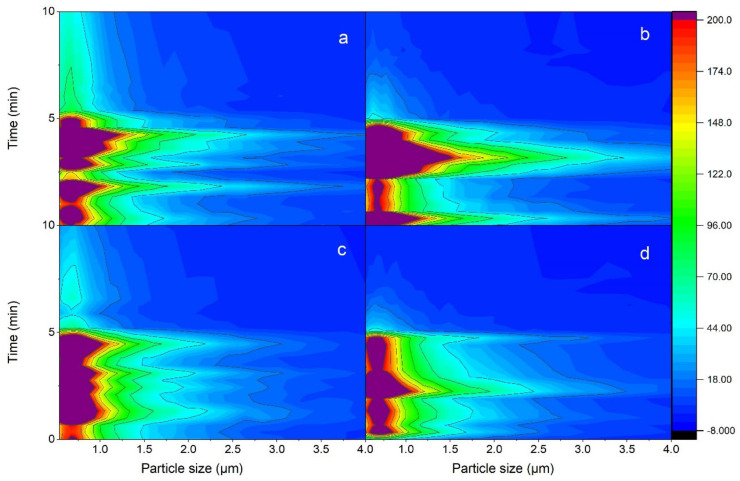
Concentrations of particles from 0.5 to 4 μm in the first 10 min measurement with (**a**,**b**) closed door with (**a**) air purifier off and (**b**) high-speed air purifier turned on from the beginning of particle generation; and (**c**,**d**) open door with (**c**) air purifier off and (**d**) high-speed air purifier turned on from the beginning of particle generation.

Figure 3 shows the incremental concentrations, which are defined as the differences between real concentrations and the background during the five min of continuous dental operation and five min afterward for cases 1, 4, 5, and 6. The concentration distribution over 10 min shows the increase in concentration in 5 min and then the decrease to lower values, which shows removal and dispersion of them from the generation zone. Figure 3 illustrates the concentration for the particle size range of 0.5 to 4 µm, while the larger size concentration (4 to 20 µm) was negligible for these cases. The color scale defines number concentrations from 0 (blue) to 200 cm^3^ (red). The values between these limits are mapped by blue, green, yellow, and orange. The purple shows values greater than 200 cm^3^. As expected, the number concentration distribution varies with the operating conditions. For all cases, the smaller the particle size, the higher the concentration.

In closed-door cases, by comparing the case in which no air purifier is running (Figure 3a) with the case in which the air purifier is running at the beginning of operation (Figure 3b), it can be observed that particles have a wider distribution in Figure 3b, which means particles are growing to larger sizes. For instance, a concentration of higher than 200/cm^3^ is observed for 0.5–1.3 μm particles in Figure 3a, while this range of concentration is observed for 0.5–1.5 μm particles in Figure 3b. Moreover, the concentration of 200–70/cm^3^ is detected for 1.3–2 μm particles in Figure 3a; however, 1.5–2.8 μm particles have this concentration range in Figure 3b. The real generated values for Figure 3b are even more than this reported number because the removal process was started at the beginning, and a fraction of particles was spread in the room during the first 5 min. Similar behavior was observed when the door was open. Table 3 lists the size distributions in 5-min particle generation corresponding to Figure 3 for the three concentration ranges of >200 (1/cm^3^), 200–70 (1/cm^3^), and 70–18 (1/cm^3^).

From this observation, it can be inferred that running the air purifier from the beginning leads to air circulation in the room. The air circulation can enhance the interaction between airborne particles in the area that particles are generated. These interactions lead to the agglomeration of 10 microns or smaller particles [38]. Thus, the particles may have grown to larger ones when the air purifier was on at the beginning of the operation. Growing to larger sizes is preferable in terms of particle removal. Removal by HEPA filter is size-dependent: the larger the sizes, the more probable filtration is. The filtration of micron particles is due to interception and impaction mechanisms [39].

The particles generated in the 5 min long operation gradually spread in the room, and their concentrations were decreased by different mechanisms. They are introduced in the next sections.

### 3.2. Temporal Change of Particle Concentrations in the Generated Zone

The temporal change of 0.5-μm particle concentrations in the generation zone is discussed in Section 3.2.1. The smaller particles probably carry more infectious microorganisms because of their high concentrations. Similar qualitative results were observed for other sizes than 0.5 μm. However, particle size is an important parameter in particle removal, which is elaborated in Section 3.2.2.

#### 3.2.1. Effects of Air Purifier and the Door Condition on the Removal of 0.5 μm Particles

**Figure 4:**
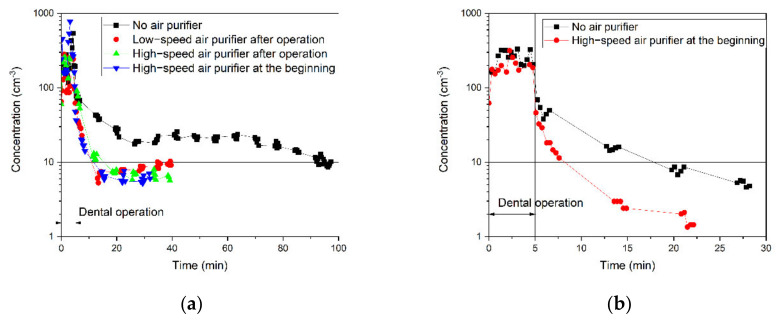
Temporal change of particle concentrations of 0.5-μm particle in the generated zone in (**a**) closed-door and (**b**) open-door cases.

Figure 4 shows real-time number concentrations of 0.5-μm particles during the dental operation and afterward until they reached the background level. Figure 4a is for the closed-door and Figure 4b for the open-door cases. The solid horizontal line marks the background concentration of 0.5-μm particles. The particle concentrations dropped gradually, likely by settlement on the surface [40], filtration by the air purifier, or dispersion in and out of the room. Table 4 summarizes the times it takes for the number concentrations to reach their background levels (removal times) for all six cases. In the worst-case scenario, when the door is closed and no air purifier is running in the room, it takes 95 min for 0.5-μm particles to return to the background level.

Two conclusions can be drawn from the results in Figure 4. First, as shown in both Figure 4a,b, the air purifier expedited particle removal from the air. For instance, Figure 4a shows that running a high-speed air purifier enhanced the removal time of 0.5-μm particles at least 6.3 times faster than the case with no air purifier. Figure 4a shows the lowest particle concentration in the room when the high-speed air purifier is running from the beginning of the operation. However, the removal time is almost the same for all these three cases: low-speed air purification after the dental operation, high-speed air purification after the dental operation, and high-speed air purification from the beginning of the operation. This observation might be because of high air changes per hour (ACPH) of 7.23 and 14.73 in the tested room with low and high fan speed, respectively. During the time that the concentration of the generation zone reaches the background, the contaminated air in this zone was taken into the air purifier at least 4.8 times with low speed (40 min,) and particles are captured by the filter. The filtration efficiency of the air purifier filter is >99.99% for 0.1–5 µm particles. Therefore, running the air purifier with both fan speeds can clean the generation zone.

It can be inferred that particles were captured with the HEPA filter and Activated Carbon Filter installed in the air purifier (from the air purifier specification). In addition to filtration, enhancing air circulation in the room by the air purifier leads to faster particle settlement on the surface areas. These results suggest that the air purifier has a crucial role in removing airborne contamination of dental offices in the generation zone.

Second, comparing the removal times of open-door cases (Figure 4b) with closed-door cases (Figure 4a) shows that opening the door expedited the removal of 0.5-μm particles in the generation zone. The open door enables the dispersion of airborne particles by natural ventilation and air circulation. Dispersed particles due to the air circulation may settle on the indoor surfaces based on impaction and interception or exit the room when a door is opened. All these mechanisms are counted as air circulation because differentiating between them is not possible in the room. Furthermore, the transport of particles through space based on air circulation is much more influential compared to their transport close to the surfaces, which leads to collision by impaction and interception. This transport due to air circulation may lead to sequential contact with the surfaces; therefore, deposition impaction and interception is almost 100% for micron-sized particles. It implies that the number concentration in the hallway was lower than inside the test room at the time of these measurements. On the other hand, external particles may enter the room and worsen the inside air quality if there are more particles outside of the door. This was the case on another day of measurement (see Section 3.4), when the dentist was operating on patients. The results of concentration measurement in three dental operations with real patients show that the concentration peaks were observed in the moments that the door was open, and the higher concentrations entered the room from outside. Therefore, opening the window, similar to the open-door cases, is recommended as a short-term solution for dental offices without air-filtration systems.

It should be noted that the recognized mechanisms for each case are the most dominant ones; however, other mechanisms might be present. For example, particles might be lost through the door, which is not perfectly sealed, such as in case 1.

The particle-removal time varies with particle size, although the air purifier and open-door condition help reduce the concentration of all sizes of particles in the generation zone. The next section elaborates on the size dependency of particle spread and removal because smaller particles probably carry more infectious microorganisms because the concentration of smaller particles is higher than the larger ones.

#### 3.2.2. Effects of Particles Size on Particle Removal

**Figure 5:**
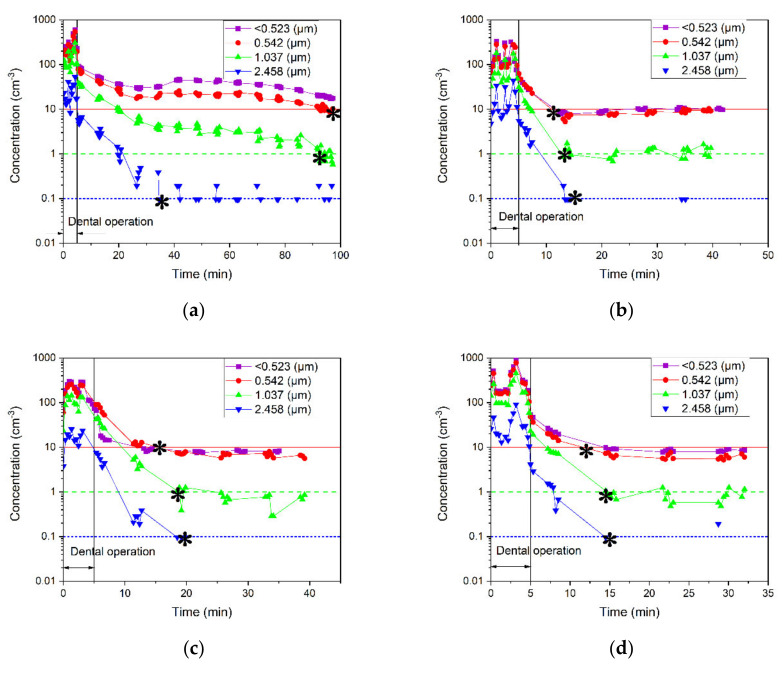

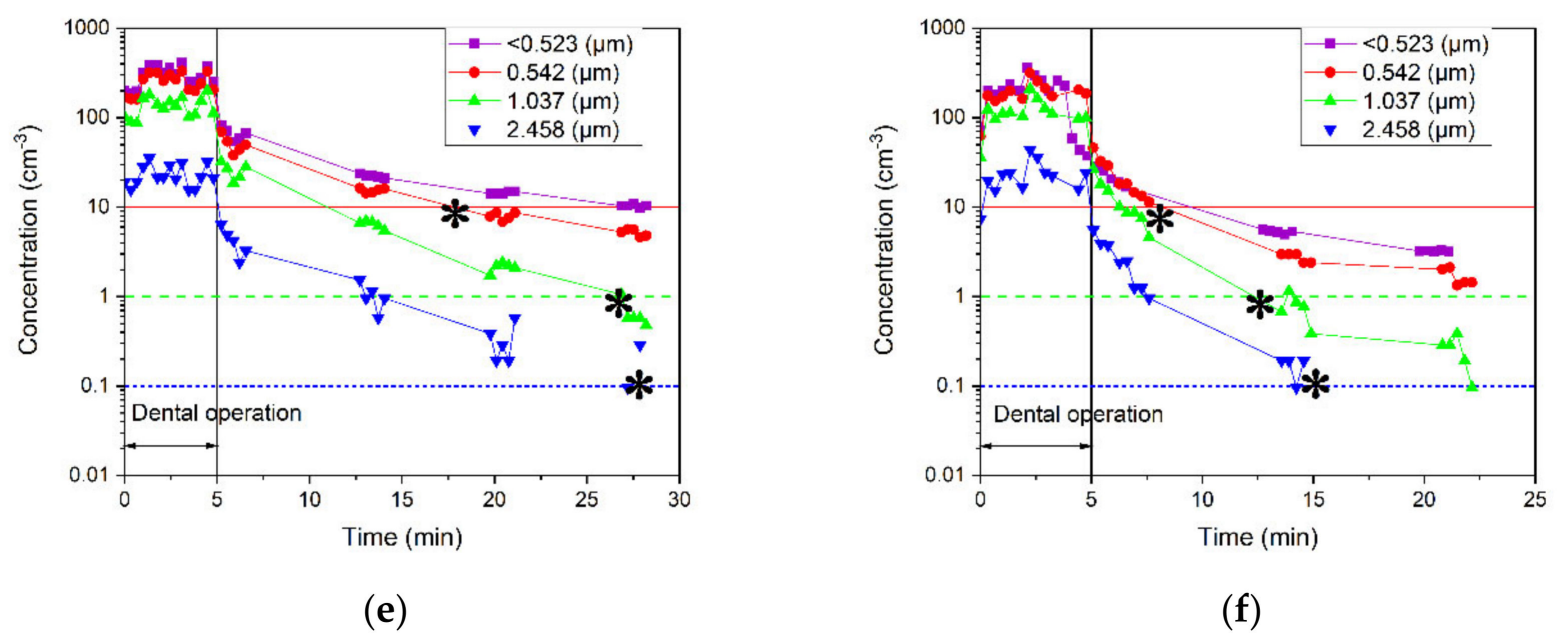
Number concentrations of sub-0.5-, 0.5-, 1-, and 2.5-μm particles measured in the generation zone for (**a**–**d**) closed-door cases with (**a**) air purifier off, (**b**) low-speed air purifier turned on after particle generation, (**c**) high-speed air purifier turned on after particle generation, (**d**) and high-speed air purifier running from the beginning of particle generation; and (**e**,**f**) open-door cases with (**e**) air purifier off and (**f**) high-speed air purifier running from the beginning of particle generation. The background concentration is shown with the red horizontal line for <0.5- and 0.5-μm particles, green dash line for 1-μm particles, and blue dotted line for 2.5-μm particles. The removal time for different particle sizes is marked with asterisks.

Figure 5 demonstrates the number concentrations of particles with sizes of sub −0.5, 0.5, 1, and 2.5 μm for all six cases. The removal times for different particle sizes are listed in Table 4.

Several mechanisms lead to particle removal from the air, including settling, air circulation, and air filtration. First, all particles in a closed-door room settle down because of gravity without major air circulation or filtration. Figure 5a shows that gravity settling is the most effective mechanism in case 1. For example, 2.5-μm particles disappeared faster than those that were smaller. It is well known that the larger particles have higher gravitational settling velocities and that their removal times are shorter than the smaller particles. Second, air circulation leads to the dispersion of particles and their subsequent removal by settling on the surface areas due to impaction and interception or exiting the room or both. The drag force on a particle is also size dependent, which is higher for a larger particle [41]. Therefore, it usually takes a longer time for a larger particle to disperse than for smaller ones. Figure 5e indicates that air circulation through the open door expedited the particle removal, although the air purifier was off. In addition, Figure 5e shows expedited removal of smaller particles and confirms that air circulation is the dominant mechanism in this case. Third, the filtration efficiency is also size dependent, and it increased with the particle size for micron particles [39].

Figure 5f for the high-speed air purifier running from the beginning of operation in the open-door room shows the combined effects of all three mechanisms. Air circulation may be the dominant size-dependent mechanism that leads to removal, although filtration also plays a significant role in the removal of all particles regardless of their size because it took longer time to reduce the concentrations of 2.5-μm particles than the smaller ones. The filtration efficiency of the installed filter in the air purifier is >99.99% for 0.1–5 µm particles, which is based on impaction and interception mechanisms.

It is also can be concluded that the removal time is also as good as the rate of removal because the mechanisms that lead to particle removal are not concentration dependent. Increasing the concentration of particles in the room does not affect the removal time of particles that are settled by inertia. All particles of the same size are settled at the same rate regardless of particle concentration. The spread of particles and their subsequent removal due to air circulation are not affected by the concentration of particles. The air velocity is not changed with particle concentration because concentration around the room is not high enough to impact the air circulation. Furthermore, the viscous drag force on particles due to the friction against air circulation in the room does not vary with the concentration of particles. With the >99.99% filtration efficiency of the filter, filtration in the room is not concentration dependent because the particles are not returned to the air. As the particles enter the air purifier, they are captured by impaction and interception mechanisms. Furthermore, the fan speed is the effective parameter to import the particles into the air purifier; with respect to the air-exchange rate of the air purifier, the filtration efficiency of the filter is 100% for the duration of measurement in all cases.

Moreover, Figure 5b–d show that the removal times do not vary with particle size. Therefore, a combination of settling, air circulation, and air filtration all play roles in particle removal for these cases. Comparing these cases with that in Figure 5f demonstrates the strong effects of air circulation due to the open door.

In summary, an air purifier running at high fan speed may ensure the removal of 0.5- to 3-μm particles, while air circulation is more effective for smaller particles. Since the door of dental offices might be open frequently, an air purifier with a strong fan may help prevent cross-contamination from one room to the other through the door. Nonetheless, our study herein does not undermine the effectiveness of external high-volume evacuation (EHVE) and suction, which are often used near to the generation zone.

However, it does not mean that the room is completely cleaned even when the particle concentrations in the generation zone dropped back to the background. The particles may be transported from the source to the rest of the room. Dental staff walks around in the same room, and they often remove their masks for a short break at the corner, where there is little air circulation. It is necessary to investigate the spread of particles by analyzing the concentration in the corner of the room, and the results are presented in the next section.

### 3.3. Spatial and Temporal Change of Particle Concentrations in the Corner of the Dental Office

#### 3.3.1. Effects of Air Purifier and the Door Condition on the Spread and Removal of 0.5-μm Particles

**Figure 6:**
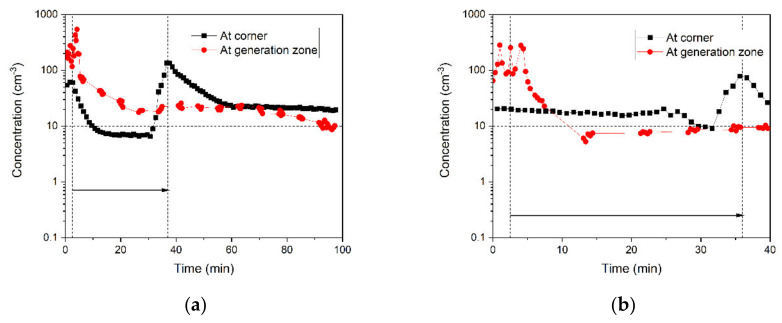

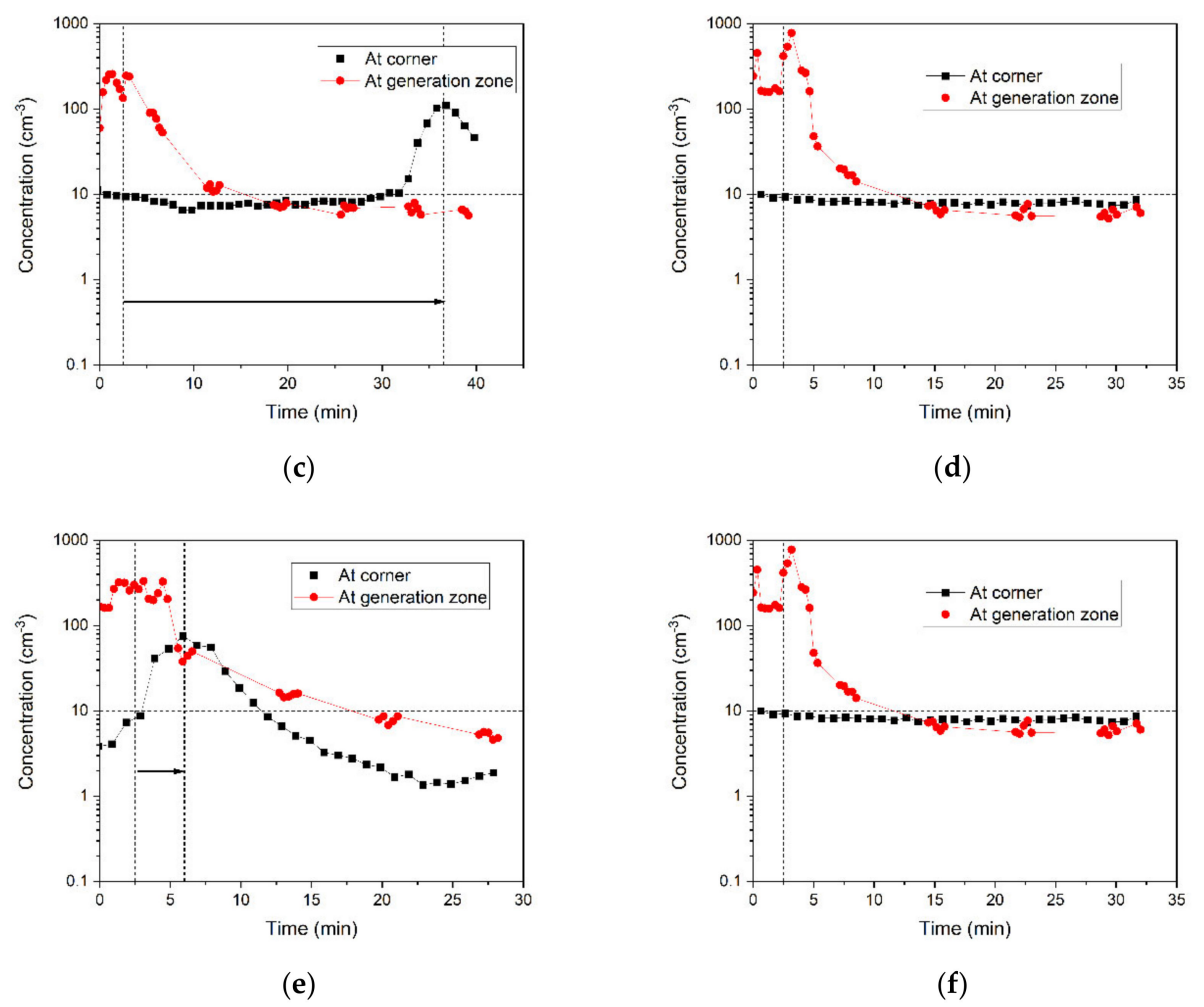
Comparison of the number concentrations of 0.5-μm particles in the corner with those at the generation zone for (**a**–**d**) closed-door cases with (**a**) air purifier off, (**b**) low-speed air purifier turned on after particle generation, (**c**) high-speed air purifier turned on after particle generation, and (**d**) high-speed air purifier running from the beginning of particle generation; and (**e**,**f**) open-door cases with (**e**) air purifier off and (**f**) high-speed air purifier running from the beginning of particle generation.

Figure 6 compares the number concentrations of 0.5-μm particles in the corner with those at the generation zone for all six cases. This comparison helps quantify the number of particles in the corner when the number concentration in the generation zone dropped to the background level. The particles moved from the generation zone to the corner for some cases. Table 5 summarizes the travel time, the time that concentration peak reaches the corner, and the peak concentration ratio, or the ratio of peak concentration at the corner to the one at the generation zone. For example, the concentration peaks are observed for all sizes in 37 min when the door was closed and the air purifier was off. In this case, the number concentration of peak in the corner is lower than the value in the generation zone. On the contrary, Figure 6d,f show that no peak is observed in the corner for 0.5-μm particles when the air purifier is running from the beginning of operation with either an open or closed door. These results indicate the effectiveness of high-speed, high-efficiency air purification running with the dental procedure. Generally, it can be inferred that the peak is observed in the corner when the rate of particle settlement and removal from the air in the generation zone is lower than particle transport to the corner.

Table 5 indicates that it took 6 min for the concentration peak to reach the corner when the door was open, and the air purifier was off. In comparison, Figure 6a shows that travel time is longer when the door was closed with the air purifier off (37 min). The air circulation resulting from the open door affected the contamination level in the room. Therefore, an open-door during operation may expedite the travel of particles from the source to the corner.

The peak concentration ratio of case 1 in which the air purifier is off in the closed-door office is 1.5 times higher than the one in case 3 in which the air purifier is running at high speed after the operation. The results indicate that the air purifier is efficient in removing particles from the generation zone by filtration and air circulation mechanisms; therefore, a lower fraction of particles travels to the corner in case 3. However, the travel time of the concentration peak is close to each other; it takes about 36 min for the remaining particles in the generation zone to transfer to the corner in both cases. This is surprising because these results imply that the air circulation result from the air purifier has little impact on the air movement to the corner of the room for all cases (See Figure 1). Many factors contribute to particle dispersion, such as particle initial velocity. The particles generated by high-speed handpieces have high initial velocity ejected from the generation zone. Therefore, the initial velocity of the particles might be a substantial factor for particle-travel time to the corner. Further air velocity measurements or CFD simulation to study the particle dispersion and the effects of air circulation caused by the air purifiers on spatial distribution could provide additional insights on particle dispersion.

#### 3.3.2. Effects of Particle Size on the Spread and Removal 

**Figure 7:**
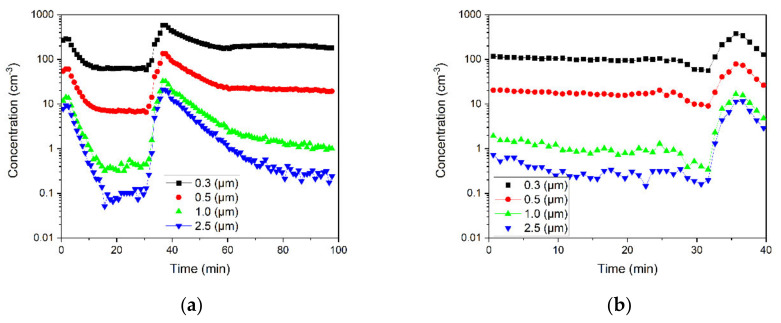

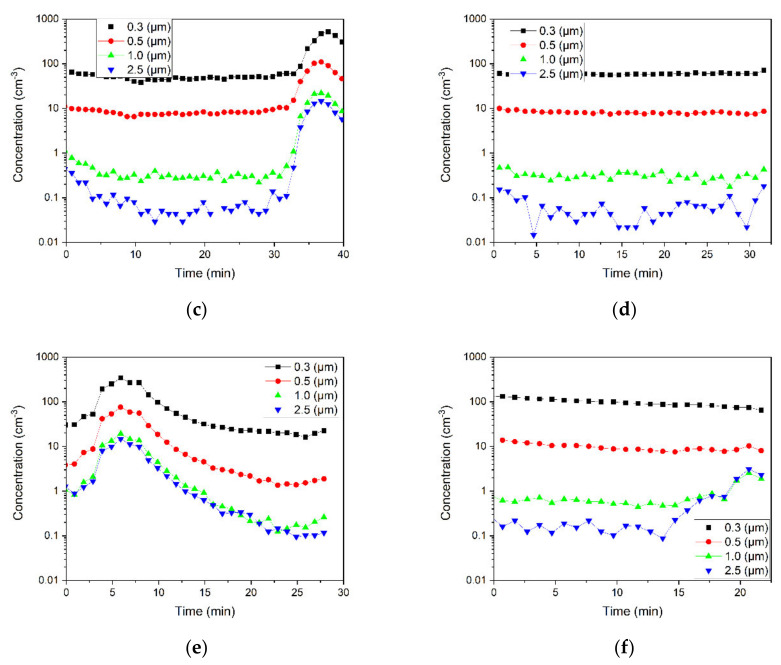
Number concentrations of sub-0.5-, 0.5-, 1-, and 2.5-μm particles measured in the corner of the office for (**a**–**d**) closed-door cases with (**a**) air purifier off, (**b**) low-speed air purifier turned on after particle generation, (**c**) high-speed air purifier turned on after particle generation, and (**d**) high-speed air purifier running from the beginning of particle generation; and (**e**,**f**) open-door cases with (**e**) air purifier off and (**f**) high-speed air purifier running from the beginning of particle generation.

Figure 7 shows the number concentrations of 0.3-, 0.5-, 1-, 2.5-μm particles in the corner of the office for all six cases. All particles reached the corner with the same travel time, as indicated by the concentration peaks observed in the corner except in one case. Figure 7f shows the concentration peaks for 1- and 2.5-μm particles but not for the 0.3- and 0.5-μm particles. This observation is expected based on two conclusions that were made in Section 3.2.2 and Section 3.3.1 for this case and confirms those earlier conclusions. First, the removal rate of larger particles is lower than the rate of smaller ones, while the air circulation due to the open door and filtration leads to particle removal. The 0.5-μm particles were removed after 8 min, while 2.5-μm particles disappeared after 15 min from the generation zone. A higher drag force on 2.5-μm particles delays their removal compared to 0.5-μm particles. In Figure 7f, the drag force is higher in comparison with other cases under the influence of air circulation through the open door and air purifier. Second, the peak is observed in the corner when the rate of particle settlement and removal from the air is lower than particle transport to the corner. Thus, only a fraction of 1- and 2.5-μm particles, which were not removed from the air, travelled to the corner.

### 3.4. Field Campaign with REAL Patients

**Figure 8:**
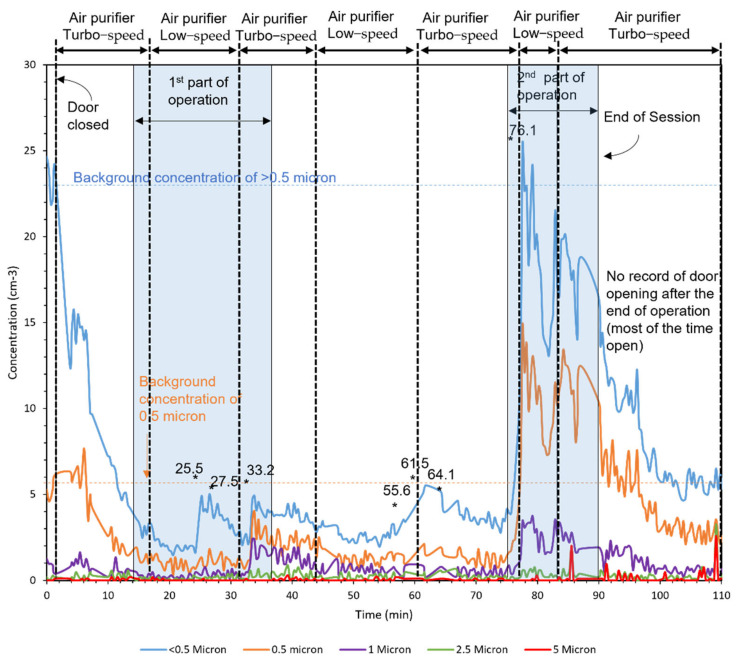
Operation 1 in room A (Large room). Asterisks in the figure represent the moments that the door was open.

**Figure 9:**
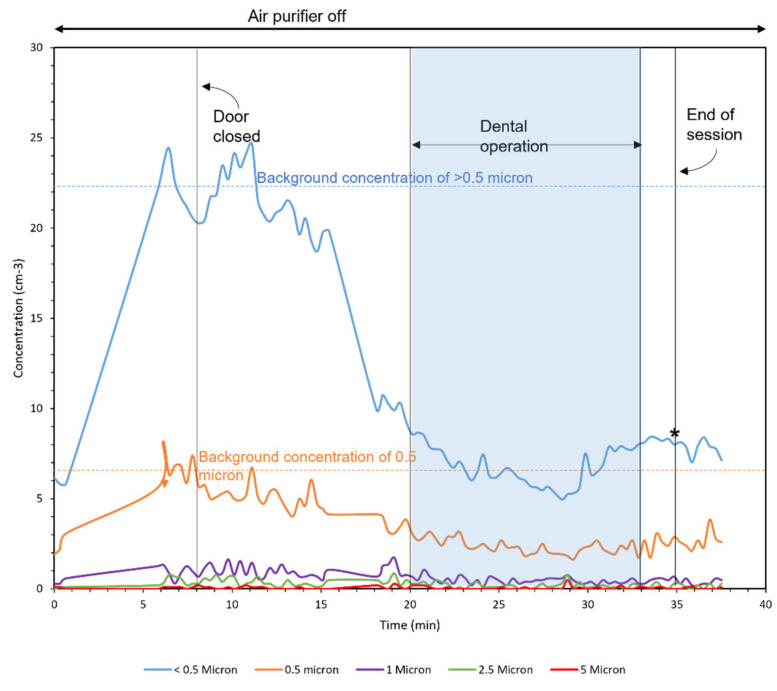
Operation 2 in room A (Large room). Asterisks in the figure represent the moments that the door was open.

**Figure 10:**
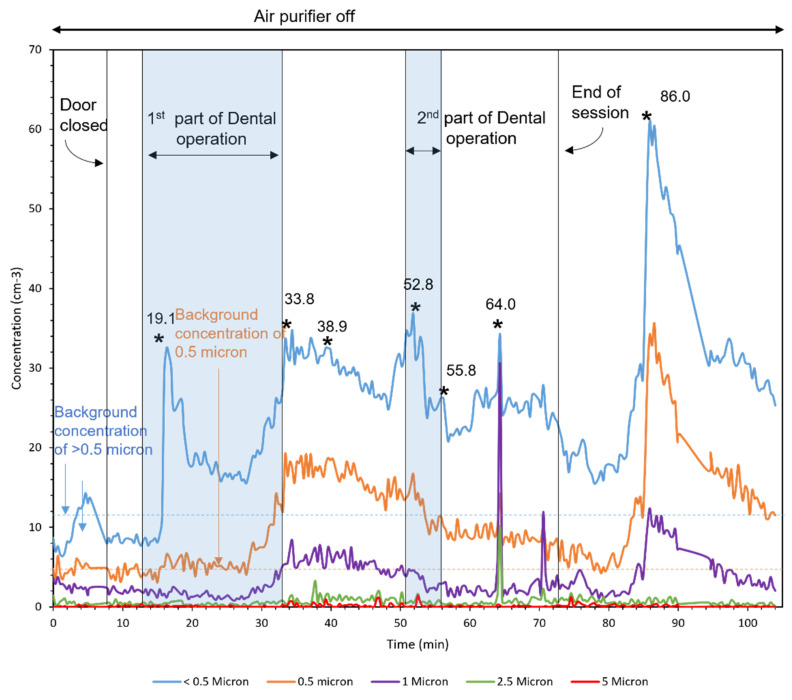
Operation 3 in room B (small room). Asterisks in the figure represent the moments that the door was open.

Figure 8, Figure 9 and Figure 10 demonstrate the number concentration of <0.5-, 0.5-, 1-, 2.5-, and 5-μm particles during three dental procedures. The duration of dental procedures was different in three cases. The horizontal lines mark the background concentrations of <0.5 and 0.5-μm particles. The moments that the door was open are shown with asterisks in the figures, which represent a duration of less than a minute.

The first operation was conducted in two parts, as shown by the patterned area. The higher concentrations entered the room from outside. After closing the door, the number concentration was reduced by the air purifier. Moreover, the concentration peaks were observed in the moments that the door was open. The major fraction of particles was generated in the second part of the operation. During this time, the air purifier was running at low speed for 7 min and turbo speed for 7 min. In the first 7 min, the removal rate was 0.28 (1/cm^3^min) and the second 7 min was 1.14 (1/cm^3^min), four times faster than the time with low speed.

The second operation was conducted in a single part, and no considerable particles were measured. Similar to the first operation, the number concentration outside was higher than that inside. The number concentration in the third operation was higher than the first two operations. The third operation was conducted in two parts. Higher values of concentration coming from outside are observed in this operation comparing to the first two because APS was closer to the door in the third operation.

## 4. Conclusions

The following conclusions can be drawn from the results of this study:

In the worst-case scenario with no protection system in the closed-door office and continuous high-speed drilling, it takes 95 min for 0.5-μm particles to return to background level and that it takes a shorter time for particles larger than 0.5 μm to be removed from the air. In the real operations with patients, which usually last less than five minutes, air may be cleaner because of other measures, like suction from the source (i.e., the mouth).

There are three size-dependent mechanisms for particle removal: gravity settling, air circulation, and air filtration. Technologies that combine all of them are the most effective in air cleaning. The air purifier expedited the removal time at least 6.3 times faster than the case with no air purifier in the generation zone. Running a high-speed air purifier at the beginning of the operation is the most effective scenario in reducing airborne particle concentrations. The air purifier at one corner could not eliminate the concentration peak in the other corner of the room except for the case when the door was closed, and the air purifier was running at the highest speed from the beginning of the operation.

When there is an indoor air purifier, it is recommended to keep the door closed during the operation; otherwise, particles may enter the hallway through the open door. These particles may transmit diseases if they carry infectious microorganisms. In dental offices without air-purification devices, it is recommended to open the window(s) when possible to promote natural ventilation; however, it may cause an accumulation of particles in a corner. In addition, staff should leave the room after the operation and close the door for particles to settle or exit the window(s). Admittedly, the surfaces should be cleaned where particles may settle on.

Our results have important implications for infectious disease transmission in closed settings, such as dentists and doctors’ offices. Although we did not study other closed environments, such as schools, our study documents the time taken for airborne particles to settle down as well as the utility of air purifiers, which highlights the importance of air circulation and filtration in closed settings. In the context of the current COVID-19 pandemic, our study findings can assist in developing guidelines for air circulation and filtration, which can significantly reduce the chances of disease transmission.

Nonetheless, further research on spatial distribution and removal of particles may provide more information to protect dental professionals, staff, and patients in dental offices.

## Figures and Tables

**Table 1:** Test cases and conditions.

Case No.	Dental Operation Duration	Door (Open/Close)	Air Purifier		
			On/Off	Fan Speed	Air Cleaning Starting Time
1	5 min	Close	Off	-	-
2	5 min	Close	On	Low (153 CFM)	After 5 min of operation
3	5 min	Close	On	High (312 CFM)	After 5 min of operation
4	5 min	Close	On	High (312 CFM)	At the beginning of the operation
5	5 min	Open	Off	-	-
6	5 min	Open	On	High (312 CFM)	At the beginning of the operation

**Table 2:** Dental operation conditions for tests with real patients in room.

Operation No.	Dental Operation		Room Size	Air Purifier	Room Condition before the Operation		Room Condition after Operation	
	Type of Operation	Duration			Temperature (°C)	Humidity (%)	Temperature (°C)	Humidity (%)
1	Filling with 1 root canal High/low-speed handpieces	88 min	Large (Room A)	On	21.9	60	23.6	58
2	Filling with 1 root canal High/low-speed handpieces	33 min	Large (Room A)	Off	24.7	54	24.9	49
3	Crown insertion High/low-speed handpieces	73 min	Small (Room B)	Off	23.3	51	24.3	51

**Table 3:** Concentration distribution in 5-min particle generation.

Case No.	Concentration >200 (1/cm^3^)	Concentration 200–70 (1/cm^3^)	Concentration 70–18 (1/cm^3^)
1	0.5–1.3	1.3–2	2–3.7
4	0.5–1.5	1.5–2.8	2.8–4
5	0.5–1	1–1.5	1.5–3
6	0.5–1.4	1.4–2	2–3.5

**Table 4:** Removal times of the cases at the generation zone.

No.	Door	Air Purifier			Removal Time at Generation Zone (min)		
		On/Off	Fan Mode (Flow Rate)	Air Cleaning Starting Time	0.5 μm	1 μm	2.5 μm
1	Close	Off	-	-	95	92	35
2	Close	On	Low (153 CFM)	After dental operation	11	13	15
3	Close	On	High (312 CFM)	After dental operation	15	18.5	20
4	Close	On	High (312 CFM)	At the beginning of the dental operation	12	14.5	15
5	Open	Off	-	-	18	26.5	28
6	Open	On	High (312 CFM)	At the beginning of the dental operation	8	12.5	15

**Table 5:** The travel time and concentration ratios of 6 cases at the corner of the dental office.

No.	Door	Air Purifier			Travel Time (min)			Concentration Ratio		
		On/Off	Fan Mode (Flow Rate)	Air Cleaning Starting Time	0.5 μm	1 μm	2.5 μm	0.5 μm	1 μm	2.5 μm
1	Close	Off	-	-	37	37	37	0.5	0.16	0.66
2	Close	On	Low (153 CFM)	After dental operation	36	36	36	0.33	0.1	0.4
3	Close	On	High (312 CFM)	After dental operation	36.5	36.5	36.5	0.33	0.11	0.5
4	Close	On	High (312 CFM)	At the beginning of the dental operation	-	-	-	-	-	-
5	Open	Off	-	-	6	6	6	0.26	0.11	0.5
6	Open	On	High (312 CFM)	At the beginning of the dental operation	-	21	21	-	0.016	0.06

## Data Availability

Data sharing not applicable.
